# *In vivo* endoscopic visualization of the Micra AV2 retrieval interface

**DOI:** 10.1093/ehjcr/ytag399

**Published:** 2026-05-29

**Authors:** Akira Yoshida, Koji Hashimoto, Takehisa Nishiguchi, Minoru Ichikawa

**Affiliations:** Department of Cardiology, Higashiosaka City Medical Center, 3-4-5 Nishi-Iwata, Higashiosaka, Osaka 578-8588, Japan; Department of Cardiology, Higashiosaka City Medical Center, 3-4-5 Nishi-Iwata, Higashiosaka, Osaka 578-8588, Japan; Department of Cardiology, Higashiosaka City Medical Center, 3-4-5 Nishi-Iwata, Higashiosaka, Osaka 578-8588, Japan; Department of Cardiology, Higashiosaka City Medical Center, 3-4-5 Nishi-Iwata, Higashiosaka, Osaka 578-8588, Japan

**Keywords:** Leadless pacemaker, Intravascular endoscopy, Leadless pacemaker retrieval

## Case description

**Figure 1:**
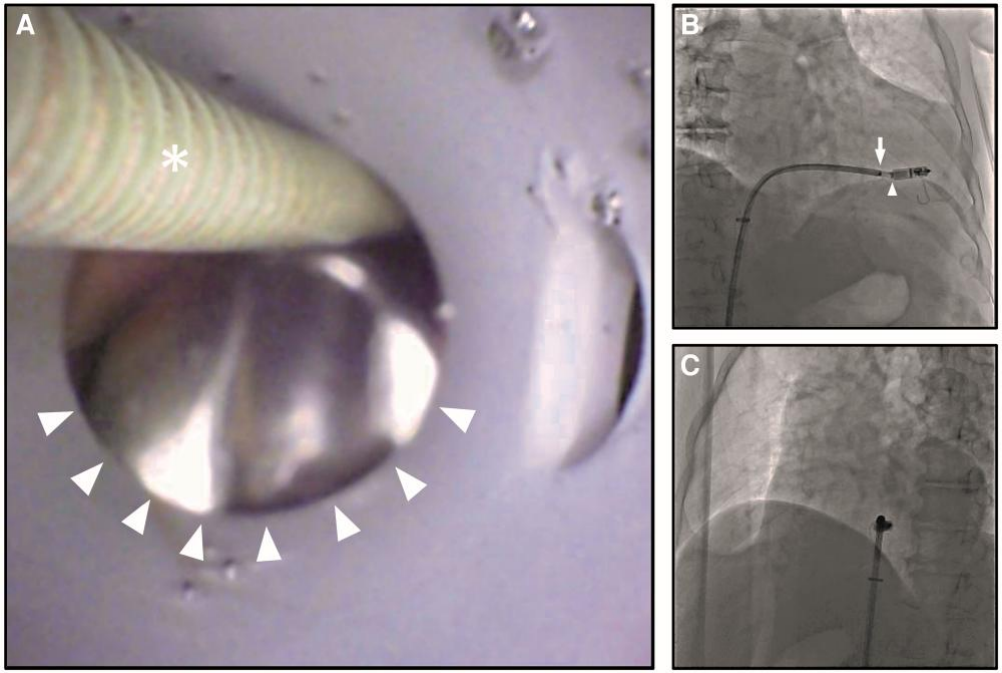
(*A*) Intravascular endoscopic view (ZEMPORSHE) of the Micra AV2 retrieval head immediately after implantation, demonstrating a distinct metallic sheen. Arrowheads indicate the retrieval head; asterisk (*) indicates the 0.035-in guidewire. (*B*) Representative still frame from fluoroscopic cine in right anterior oblique projection demonstrating proximity between the Agilis sheath tip and Micra retrieval head. The arrow indicates the endoscope tip; the arrowhead indicates the retrieval head. (*C*) Fluoroscopic cine still image in left anterior oblique projection showing coaxial alignment between the Agilis sheath and implanted Micra AV2 device.

A 76-year-old man with high-grade atrioventricular block underwent implantation of a Micra AV2 leadless pacemaker (Medtronic, Minneapolis, MN, USA) based on patient preference to avoid a transvenous pacing system. Following successful deployment, final electrical parameters were as follows: capture threshold, 0.63 V at 0.24 ms; R-wave amplitude, 14.0 mV; and impedance, 720 Ω. Intravascular endoscopy (ZEMPORSHE; OVALIS, Osaka, Japan) was performed immediately after implantation to visualize the device–tissue interface. The endoscope was advanced through an Agilis L-curve steerable sheath (Abbott, Abbott Park, IL, USA) positioned within the Micra introducer sheath; a 0.035-in guidewire and the endoscope were inserted through this system under fluoroscopic guidance. Endoscopic imaging provided direct visualization of the Micra retrieval head with a distinct metallic sheen (*Figure 1A*; Supplementary material online, *Video S1*). Fluoroscopic cine imaging demonstrated proximity between the Agilis sheath tip and Micra retrieval head. The right anterior oblique projection still images show the endoscope tip (arrow) and retrieval head (arrowhead) (*Figure 1B*). The left anterior oblique projection confirmed coaxial alignment between the Agilis sheath and Micra implanted device (*Figure 1C*). The procedure was completed without complications, and pacing parameters remained stable following endoscopy.

Percutaneous Micra removal is most frequently performed early after implantation with high success rates.^1^ However, postmortem data suggest that fibrous encapsulation can be variable and not strictly time-dependent, and that the retrieval knob may or may not be covered by fibrous tissue.^2^ Because the objective *in vivo* assessment of retrieval-interface coverage remains limited, direct endoscopic visualization of the retrieval head before planned retrieval may provide valuable information for procedural planning in leadless pacemaker retrieval. This approach has potential limitations including additional cost and the learning curve associated with endoscopic manipulation. Although no complications were observed in this case, manipulation of the steerable sheath within the delivery system could theoretically result in device dislodgement due to inadvertent contact with the Micra retrieval feature.

## Supplementary Material

ytag399_Supplementary_Data

## Data Availability

The data underlying this article are available in the article and its online supplementary material. Additional data will be shared on reasonable request to the corresponding author, subject to ethical and privacy considerations.

## Notes

**Consent:** Written informed consent for publication of the images and accompanying videos were obtained from the patient.
